# Debate: differences and similarities between tension-type headache and migraine

**DOI:** 10.1186/s10194-023-01614-0

**Published:** 2023-07-21

**Authors:** Dilara Onan, Samaira Younis, William David Wellsgatnik, Fatemeh Farham, Saulius Andruškevičius, Ana Abashidze, Asel Jusupova, Yuriy Romanenko, Oxana Grosu, Meerimgul Zamirbekovna Moldokulova, Ulkar Mursalova, Saida Saidkhodjaeva, Paolo Martelletti, Sait Ashina

**Affiliations:** 1 Spine Health Unit, Faculty of Physical Therapy and Rehabilitation, Institute of Health Sciences, Hacettepe University, Ankara, Turkey; 2 Danish Headache Center, Department of Neurology, Rigshospitalet Glostrup, Copenhagen, Denmark; 3 Department of Clinical and Molecular Medicine, Sapienza University, Rome, Italy; 4 Department of Headache, Iranian Centre of Neurological Researchers, Neuroscience Institute, Tehran University of Medical Sciences, Tehran, Iran; 5 Center of Neurology and Center of Anesthesiology, Intensive Care and Pain Management, Vilnius University Hospital SantarosKlinikos, Vilnius, Lithuania; 6 Department of Neuroscience, Caucasus Medical Centre, Tbilisi, Georgia; 7 Department of Neurology and Clinical Genetics, Kyrgyz State Medical Academy, Bishkek, Kyrgyzstan; 8 Medical Center “Medical Ventera Group”, Kyiv, Ukraine; 9 Diomid Gherman Institute of Neurology and Neurosurgery, Headache Center, Chisinau, Moldova; 10 I.K.Akhunbaev Kyrgyz State Medical Academy, Bishkek, Kyrgyzstan; 11 Republican Perinatal Center, Baku, Azerbaijan; 12 Department of Neurology, Child Neurology and Medical Genetics, Tashkent Pediatric Medical Institute, Tashkent, Uzbekistan; 13 Department of Neurology and Department of Anesthesia, Critical Care and Pain Medicine, BIDMC Comprehensive Headache Center, Harvard Medical School, Beth Israel Deaconess Medical Center, Boston, MA USA; 14 Department of Clinical Medicine, Faculty of Health Sciences, University of Copenhagen, Copenhagen, Denmark

**Keywords:** CGRP, Central sensitization, Headache, International Classification of Headache Disorders, Migraine, Pericranial tenderness, Tension-type headache, Triptan

## Abstract

Tension-type headache (TTH) and migraine are two common primary headaches distinguished by clinical characteristics according to the 3^rd^ edition of the International Classification of Headache Disorders. Migraine is identified by specific features such as being more prevalent in females, being aggravated by physical activity, certain genetic factors, having photophobia, phonophobia, nausea, vomiting, or aura, and responding to specific drugs. Nonetheless, TTH and migraine share some common characteristics, such as onset occurring in the 20 s, and being triggered by psychological factors like stress, moderate pain severity, and mild nausea in chronic TTH. Both conditions involve the trigeminovascular system in their pathophysiology. However, distinguishing between TTH and migraine in clinical practice, research, and epidemiological studies can be challenging, as there is a lack of specific diagnostic tests and biomarkers. Moreover, both conditions may coexist, further complicating the diagnostic process. This review aims to explore the similarities and differences in the pathophysiology, epidemiology, burden and disability, comorbidities, and responses to pharmacological and non-pharmacological treatments of TTH and migraine. The review also discusses future research directions to address the diagnostic challenges and improve the understanding and management of these conditions.

**Graphical Abstract:**

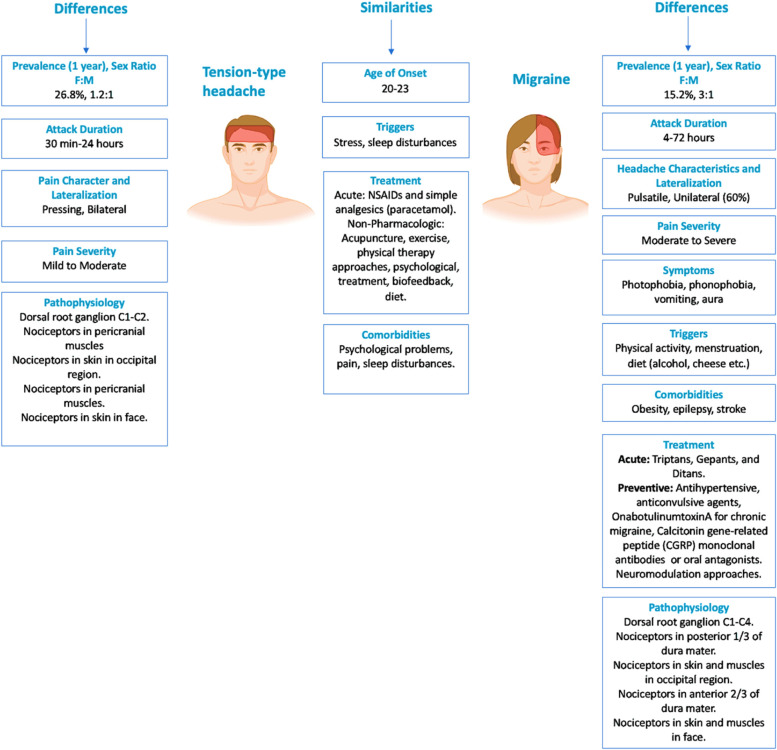

## Introduction

Headache disorders are prevalent neurological conditions and are estimated to affect around 50% of the general population (https://www.who.int/news-room/fact-sheets/detail/headache-disorders) [1]. Tension–type headache (TTH) and migraine are the two most prevalent primary headache disorders. While the International Classification of Headache Disorders (ICHD-3) [2] differentiates these headache conditions based on clinical characteristics, there is ongoing debate regarding the similarities and differences. The challenges associated with distinguishing TTH from migraine in clinical practice, clinical research, and epidemiological studies have been widely recognized [3, 4]. As there are currently no specific diagnostic tests and biomarkers available, the diagnosis of most primary headache disorders continues to rely solely on clinical assessment, making the diagnostic process complicated. This can result in clinicians mistakenly diagnosing a patient with migraine when they are actually experiencing TTH, and vice versa [3, 5, 6]. Moreover, the coexistence of TTH and migraine can add further complexity to the diagnosis. The Spectrum Study [6] found that 32% of patients who were initially diagnosed with TTH were later diagnosed with migraine or migrainous headache based on a neurologist’s evaluation of headache diaries and medical records kept for up to 6 months after the initial diagnosis. Of note, the current ICHD-3 diagnostic criteria for chronic migraine permit patients to have TTH-like headache [2]. This review aims to discuss the similarities and differences between TTH and migraine, with reference to current literature.

### Epidemiology

According to epidemiological studies, tension-type headache (TTH) has a higher prevalence than migraine. The global 1-year prevalence for TTH is estimated to be 26.8%, while the global 1-year prevalence for migraine is 15.2% in the general population [7, 8]. It has been observed that there is no difference in the prevalence of TTH among individuals with migraine compared to individuals in the general population [8]. However, the frequency and severity of headache attacks may be higher in individuals with TTH coexistent with migraine when compared to the attacks experienced by individuals with TTH without migraine [8]. Migraine is more prevalent in females, with a female-to-male ratio of 3:1 [5]. In contrast, the prevalence in TTH is more equally distributed with a female-to-male ratio of 1.2:1, possibly indicating that hormonal factors play a bigger role in migraine than in TTH. The onset of migraine is reported by about 75% of individuals before the age of 35 years [9]. The onset of migraine may begin at any age, but it typically occurs during puberty and adolescence, and rarely after 50 years of age [10]. In comparison, the age of onset is overall lower in TTH than in migraine (< 30 years vs > 30 years) [11]. However, the prevalence of both migraine and TTH peaks between ages 35–39 years, followed by a decline [10].

One population study conducted on school-age children has reported that the 1-year prevalence of migraine was 7% [12]. In a recent review, the prevalence of both migraine (5%) and TTH (5.9%) in children (0–9 years) was reported comparably low, with no difference between boys (~ 5%) and girls (~ 6%) [7]. In adolescents (10–19 years), the prevalence increases, particularly in girls than in boys with migraine (15.3% vs 10.2%) (15.3% vs 10.2%). In contrast, the prevalence is similar between boys and girls with TTH (27.1% vs 25.5%). The review also showed that the prevalence of migraine declined (7.9%) in individuals above 65 years of age, while the prevalence of TTH further increased (32.2%) at age above 65 years (based on a single study in those above 64 years of age). These findings were in contrast to the Global Burden of Disease study, which found that the prevalence of TTH keeps declining with increasing age, including past 65 years of age, after peaking between ages 35–39 years [10].

In a 12-year longitudinal Danish population-based study, several risk factors were identified for migraine, including familial disposition, no vocational education, a high workload, and frequent TTH [13]. TTH was found to have different risk factors, including young age, female sex, poor self-rated health, not being able to relax after work, and sleeping a few hours per night. High migraine attack frequency and young age at onset (< 20 years) were found to be related to poor outcomes in migraine [14], defined as having > 14 migraine days/year either due to increased frequency or persisting high frequency of migraine. Poor outcomes in TTH were associated with baseline chronic TTH, coexisting migraine, being unmarried and sleeping problems are associated with poor outcomes in TTH [14]. In addition, poor outcome of pure TTH was associated with unilateral headache, nausea, and individual headache attack duration > 72 h, while migraine (with or without coexistent TTH) was associated with a baseline pulsating quality, severe pain intensity, photophobia, and phonophobia as well as longer duration of an individual headache attack [15].

### Burden-impact-disability

Both migraine and TTH have a substantial impact on individuals and society. These primary headache disorders affect individuals at a younger age and potentially during the most productive period of their lives. According to the findings of the systematic review conducted by the Global Burden of Disease (GBD) in 2019, headache disorders are among the top ten for disability-adjusted life years (DALYs) for both females and males in the age range of 10–49 years [16]. The high number of people who suffer from migraines and TTH contributes to the burden associated with all headache disorders. In terms of years lived with a disability (YLD), migraine remains at the top of the list with 754/100,000 age-standardized YLD rates in 2019, while TTH is responsible for only 73.9/100,000 age-standardized YLDs. Both diseases have shown an increase of 7.7% and 4.1%, respectively, since 1990 [17].

In 2021, using data from population-based studies in nine different countries, it was found that there were positive correlations between migraine disability and lost paid work time was found with a statistical significance variation of < 0.05 to < 0.001 between some countries. Additionally, the association between migraine-attributed disability and lost household work time or total lost productivity (paid + household) was highly significant (*p* < 0.001) in almost all countries studied. The authors of the study emphasized the importance of appropriate treatment and suggested that it is reasonable to expect that migraine sufferers could recover more than twenty percent of their lost productivity [18]. A Europe-wide analysis has revealed that the average direct cost of episodic migraine (EM) was €746 per year, whereas the average direct cost of chronic migraine (CM) was €2,427 per year [19, 20]. In the United States, these costs are around three times higher [19, 21]. According to the Danish study, the number of work days lost due to TTH was 820 and the number of work days lost due to migraine was 270 per 1,000 individuals per year, indicating a greater burden of TTH [5, 22]. Studies indicate that migraine is associated with higher economic resource use and medical costs in outpatient, inpatient, and emergency admissions compared to TTH. In fact, this situation is exacerbated by the chronicity of the disease. In a 2002 study investigating the loss of productivity, it was reported as 5431 for migraine (3565 lost work days) and 2795 for TTH (1523 lost work days) in US dollars in the last 4 weeks before assessment [23]. In another study in 2020, it was stated that individuals experienced the loss of work days with a rate of 7.1% in migraine and 2.2% in TTH [24]. Therefore, there are opinions that migraine creates a greater burden compared to TTH, particularly in terms of lost work days and economic costs.

### Comorbidities

Both migraine and TTH are associated with comorbidities, although there are differences in the relative frequency of some of the comorbidities between the two headache types. These differences may have clinical and therapeutic implications. Anxiety and depression are more prevalent in primary headache than in the non-headache population [25–27]. Additionally, anxiety and depression positively correlate with the frequency and intensity of headaches [28–30]. The relationship between psychiatric comorbidities and primary headaches comorbidity has been reported to be bidirectional [26, 31–33]. There is a higher prevalence of depression and anxiety in migraine (6.9% and 19.1%) than in TTH (4.5% and 12.1%) [27]. Depression in migraine is associated with an increased risk of chronification, which increases with depression severity [34]. The frequency of neck pain (NP) (90%) and low back pain (LBP) (80%) in TTH was shown to be high [35]. The headache frequency positively correlates with the frequency of NP in TTH [35, 36]. NP is 12 times more prevalent in migraine compared to non-headache controls and two times more prevalent in those with CM compared to EM [37]. The coexistence of TTH and migraine may influence the prevalence of NP in migraine, as pericranial tenderness in TTH may possibly increase the relative frequency of NP in migraine. One study showed relatively more frequent NP in patients with coexistent migraine and TTH than those with pure migraine [35]. The German Headache Consortium reported that low back pain (LBP) had a higher rate in CM and chronic TTH (CTTH) compared to those without headache, while LBP in EM and episodic TTH (ETTH) had a higher rate compared to those without headache. In fact, the rate in CM and CTTH was higher than the rate in EM and ETTH. An abnormal pain processing process may be associated with headaches and LBP [38]. One study showed a positive correlation between the number of days with TTH or migraine and the number of days with back pain in the past year [36]. Among comorbidities of primary headache disorders fibromyalgia is frequently encountered, with a prevalence of 26%, and occurring predominantly in the female and young population [39]. In patients with fibromyalgia, the lifetime prevalence of CTTH and migraine was estimated to be 48 and 56%, respectively [40]. This co-occurrence does not simply involve the sum of symptoms from both conditions but often results in an enhancement of symptoms from one condition (fibromyalgia) due to the triggering action of the other (migraine headache) [41]. The association between TTH and obesity is less studied. One study reported no association between adults with ETTH and obesity, and there is no difference in the frequency of headache when compared to individuals with normal weight [42, 43]. However, one study reported an increased risk of TTH in adolescents who were overweight or obese [44]. In contrast, there is a stronger association between migraine and obesity, supported by increased attack frequency and higher disability grades, suggesting body mass index (BMI)-dependent increased migraine severity [43, 45]. Higher BMI was associated with increased severity, frequency, and disability of migraine in children [46]. According to a population-based study, obesity is considered a risk factor for migraine chronification in overweight, obese, and morbidly obese groups, while for TTH, it is only a risk factor in the morbidly obese group [47]. Both sleep disturbance and stress are common migraine and TTH triggers [48–50]. The frequency of insomnia is higher in individuals with migraine compared to those without headache, and both migraine and non-migraine headache are more prevalent in individuals with insomnia compared to those without [51]. The prevalence of insomnia among individuals with TTH is higher than among those without headache [52]. Studies show the association between TTH and sleep issues, such as daytime sleepiness, insomnia, poor sleep quality, and shift work [5]. Insomnia is more prevalent in CTTH than ETTH [53] and sleep disorders may increase the risk of chronification in both TTH [53] and migraine [54]. Compared to TTH, migraine is more commonly associated with an increased risk of stroke (both ischemic and hemorrhagic) and myocardial infarction, especially in women and individuals with migraine with aura [55]. Studies also report the association between migraine and coronary procedures and cardiovascular mortality. TTH has not been associated with the risk of cerebrovascular or cardiovascular disease. Epilepsy is comorbid with migraine, likely in a bidirectional relationship, and may be due to shared genetic factors [56]. However, the relationship between epilepsy and TTH is less evident. According to cohort studies, 1–10% of epilepsy patients have pre-seizure headaches (30–60% of patients have a migraine-like headache, 20% of patients have TTH), and post-seizure headaches (50% of patients have a migraine-like headache) can be seen in 45% of epilepsy patients [56].

### Disease phenotype

Migraine and TTH can have some overlap in their clinical features (Table 1). Diagnostic characteristics of TTH and migraine are presented in Tables 2 and 3. The ICHD-3, TTH is categorized into three subtypes: infrequent ETTH, frequent ETTH, and CTTH [57]. Migraine has been categorized as migraine with and without aura. Furthermore, similar to TTH, migraine can be classified as chronic. Chronic migraine is having at least 15 headache days a month, with at least 8 days of having migraine features headaches, for more than 3 months (Table 3) [2].

**Table 1:** Clinical features of migraine and TTH

	**Migraine**	**TTH**
Lateralization	Unilateral (60%)	Bilateral
Headache characteristics	Pulsatile	Pressing
Duration	4–72 h	30 min-24 h
Severity	Moderate to severe	Mild to moderate
Aggravated by normal physical activity	+	-
Nausea or Vomiting	Common	Mild nausea in CTTH
Photophobia, Phonophobia or Aura	Common	Typically not accompanied

*TTH* Tension-type headache, *CTTH* Chronic tension-type headache

**Table 2:** ICHD-3 diagnostic criteria for the tension-type headache

**Infrequent episodic tension-type headache with/without pericranial tenderness on manual palpation**
A. At least 10 episodes of headache occurring on < 1 day/month on average (< 12 days/year) and fulfilling criteria B-D B. Lasting from 30 min to 7 days C. At least two of the following four characteristics: 1. bilateral location 2. pressing or tightening (non-pulsating) quality 3. mild or moderate intensity 4. not aggravated by routine physical activity such as walking or climbing stairs D. Both of the following: 1. no nausea or vomiting 2. no more than one of photophobia or phonophobia E. Not better accounted for by another ICHD-3 diagnosis
**Frequent episodic tension-type headache**
A. At least 10 episodes of headache occurring on 1–14 days/month on average for > 3 months (≥ 12 and < 180 days/year) and fulfilling criteria B-D B. Lasting from 30 min to 7 days C. At least two of the following four characteristics: 1. bilateral location 2. pressing or tightening (non-pulsating) quality 3. mild or moderate intensity 4. not aggravated by routine physical activity such as walking or climbing stairs D. Both of the following: 1. no nausea or vomiting 2. no more than one of photophobia or phonophobia E. Not better accounted for by another ICHD-3 diagnosis
**Chronic tension-type headache**
A. Headache occurring on ≥ 15 days/month on average for > 3 months (≥ 180 days/year), fulfilling criteria B-D B. Lasting hours to days, or unremitting C. At least two of the following four characteristics: 1. bilateral location 2. pressing or tightening (non-pulsating) quality 3. mild or moderate intensity 4. not aggravated by routine physical activity such as walking or climbing stairs D. Both of the following: 1. no more than one of photophobia, phonophobia or mild nausea 2. neither moderate nor severe nausea nor vomiting E. Not better accounted for by another ICHD-3 diagnosis

*ICHD-3* International Classification Headache Disorders, 3 rd Edition

**Table 3:** ICHD-3 diagnostic criteria for migraine

**Migraine without aura**
A. At least five attacks fulfilling criteria B-D B. Headache attacks lasting 4–72 h (when untreated or unsuccessfully treated) C. Headache has at least two of the following four characteristics: 1. Unilateral location 2. Pulsating quality 3. Moderate or severe pain intensity 4. Aggravation by or causing avoidance of routine physical activity (e.g. walking or climbing stairs) D. During headache at least one of the following: 1. Nausea and/or vomiting 2. Photophobia and phonophobia E. Not better accounted for by another ICHD-3 diagnosis
**Migraine with aura**
A. At least two attacks fulfilling criteria B and C B. One or more of the following fully reversible aura symptoms: 1. Visual 2. Sensory 3. Speech and/or language 4. Motor 5. Brainstem 6. Retinal C. At least three of the following six characteristics: 1. At least one aura symptom spreads gradually over ≥ 5 min 2. Two or more aura symptoms occur in succession 3. Each individual aura symptom lasts 5–60 min 4. At least one aura symptom is unilateral 5. At least one aura symptom is positive 6. The aura is accompanied, or followed within 60 min, by headache D. Not better accounted for by another ICHD-3 diagnosis
**Chronic migraine**
A. Headache (migraine-like or tension-type-like) on ≥ 15 days/month for > 3 months, and fulfilling criteria B and C B. Occurring in a patient who has had at least five attacks fulfilling criteria B-D for migraine without aura and/or criteria B and C for migraine with aura C. On ≥ 8 days/month for > 3 months, fulfilling any of the following: 1. Criteria C and D for migraine without aura 2. Criteria B and C for migraine with aura 3. Believed by the patient to be migraine at onset and relieved by a triptan or ergot derivative D. Not better accounted for by another ICHD-3 diagnosis

*ICHD-3* International Classification Headache Disorders, 3 rd edition

TTH is typically described as a bilateral, mild to moderate headache with a pressing or tightening quality that is not aggravated by routine physical activity [5, 58]. The headcahe is often can be described as a dull, aching pain that is felt in the forehead, temples, or back of the head, and can be described as a "hatband" or "vise-like" sensation [5, 57–59]. On the other hand, migraine usually presents as a unilateral, pulsating headache of moderate to severe intensity, although approximately 40% of patients with migraine may report bilateral headaches, and 26% report strictly unilateral headaches [60, 61]. Migraine and TTH can be triggered and aggravated by similar psychological factors such as stress, anxiety, and depressive symptoms [9, 60, 62]. Photophobia and phonophobia are more common in migraine than in TTH, with photophobia being present in over 80% of migraine patients, while nausea and vomiting are more common in migraine than in TTH, although mild nausea may be present in CTTH according to the ICHD-(3) [63, 64]. Nausea and vomiting are common symptoms in migraine but not in TTH. However, patients with CTTH may have mild nausea according to ICHD-3 [2].

Cranial autonomic symptoms are common in patients with migraine, occurring in 30–75% of cases, but are absent in patients with TTH [65–68]. Eye redness and tearing are the most commonly reported symptoms [68]. Patients with migraine commonly experience cutaneous allodynia, which can be cephalic and/or extracephalic [69, 70]. Ictal cutaneous allodynia is also frequently experienced by patients with migraine during the attack, with a relative frequency of 81.3% in episodic migraine, 92.5% in chronic migraine in clinic-based studies, and 63.2% in population studies [71, 72]. In addition, 79% of migraine patients have cutaneous allodynia when assessed with quantitative sensory testing [69]. Allodynia is more commonly reported in chronic migraine [71]. On the other hand, pericranial muscle tenderness is a common finding in TTH, particularly in CTTH [73, 74]. Both allodynia and pericranial tenderness are considered as clinical markers of central sensitization [75, 76].

Premonitory or prodromal symptoms are characteristic of migraine and include yawning, change in mood, fatigue, and neck pain. These symptoms typically occur within 2–48 h of the onset of migraine headache [55, 77, 78]. No reports exist of premonitory symptoms in patients with TTH. Migraine headaches may also be associated with menstrual periods, with changes in female sex hormone levels affecting migraine headache frequency. Migraine attacks frequently occur during the perimenstrual period and are usually improved during pregnancy [79, 80].

### Pathogenesis

#### *Genetics*

The pathophysiological mechanisms underlying migraine and TTH are complex, and while there are some similarities, there are also significant differences between these two primary headaches. Furthermore, the genetic mechanisms involved in these headaches differ as well. Genetic predisposition appears to be more evident in CTTH, with a threefold increase in the risk of inheritance in first-degree relatives of individuals with CTTH [81]. However, coexistent migraine and its possible influence on TTH genetic was not evaluated in this study [81]. Genetic factors may also be more evident in frequent ETTH, but not in infrequent ETTH [55].

Several genes have been linked to CTTH, including the serotonin transporter protein (5-HTT)-gene-linked polymorphic region (5-HTTLPR) genotype and the Val158Met COMT (encoding catechol-O-methyltransferase) polymorphism [82–84]. In migraine, genetic predisposition is stronger and accounts for 40%–60% of the cases [55]. The inheritance in migraine is mostly polygenic, with a few rare exceptions such as hemiplegic migraine (CACNA1A, ATP1A2, SCN1A) and cerebral autosomal dominant arteriopathy with subcortical infarcts and leukoencephalopathy (CADASIL) (NOTCH3) [55, 59]. One extensive genome-wide meta-analysis of migraine reported 123 migraine risk loci, with 86 newly explored [85]. Following the stratification, it was found that three risk variants appear to be specific for migraine with aura (HMOX2, CACNA1A, and MPPED2), two risk variants appear to be specific for migraines without aura (near SPINK2, and near FECH), and nine risk variants increase migraine susceptibility regardless of subtype. Genes that code for current therapeutic targets for migraines, such as calcitonin gene-related peptide (CGRP) (CALCA/CALCB) and serotonin 1F receptor, are among the new risk loci identified for the disease (HTR1F) [85]. The genetic factors are stronger in migraine with aura than in migraine without aura.

#### *Nociceptive mechanisms*

**Fig. 1:**
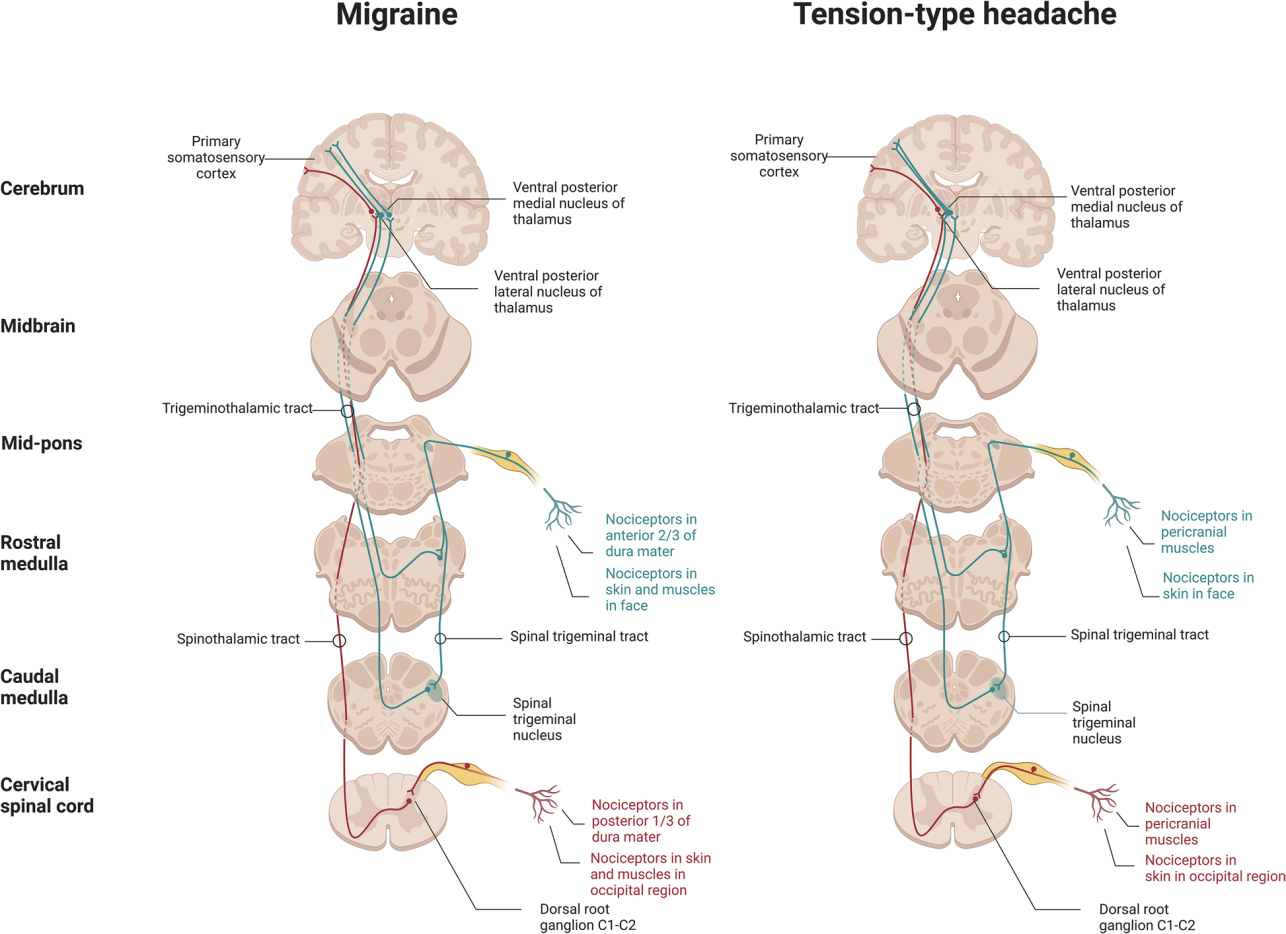
Pathophysiology of migraine and tension-type headache: nociceptive pathways

The trigeminovascular system is the anatomical and physiological substrate of TTH [5] and migraine [59] and refers to the interconnected network of nerves, blood vessels, and pain-sensitive structures in the head and neck region, including muscles (see Fig. 1). The afferent fibers of the first-order trigeminovascular neurons innervate the meninges and its blood vessels, while their cell bodies are located in the trigeminal ganglion. The ascending nociceptive input projects to second-order neurons in the brainstem, including the trigeminocervical complex, which further activates and sensitizes third-order neurons in the thalamus. From here, information is projected further to the somatosensory cortex and other cortical areas leading to the perception of pain [86].

In migraine, activation and sensitization of the first-order trigeminovascular neurons, via input from the meninges and its vasculature, plays a prominent role in the onset of the nociceptive transmission, contributing to the perception of migraine pain [59, 87]. In TTH, muscular nociceptors may be involved in the initial pathophysiological events []. The input from myofascial structures via the trigeminal ganglion and the dorsal horns of the spinal cord at levels C1 to C4 contributes to headache onset in TTH. Cerebral vascular input is of minor importance in TTH compared to migraine [5, 88]. Tenderness of pericranial muscles and tendon insertions is more common in TTH than in migraine [5, 89], and is associated with increased headache frequency (more common in CTTH vs ETTH) [73, 90], intensity, and is exacerbated during the headache phase in TTH [91]. One population-based study showed that individuals with ETTH, who developed CTTH after 12 years also experienced concomitant lower pain thresholds, suggesting an association between sensitization and chronification or transformation from episodic to chronic (≥ 15 days with headache per month) headache [92]. However, pericranial tenderness is also prominent outside the headache phase in both ETTH and CTTH when compared to controls [93, 94]. In CTTH, there is a lower cutaneous and intramuscular pain detection thresholds and tolerance thresholds in both cephalic and extracephalic regions compared with controls [5].

Research suggests that the pain detection threshold is normal or low in the cephalic region of individuals with frequent ETTH, indicating that peripheral factors are more involved in this subtype of TTH [73, 95, 96]. Thus increasing headache frequency may likely lead to central sensitization, leading to the development of CTTH [76]. It is still debated whether the pericranial muscle tenderness contributes to the development of TTH attacks or is a consequence of the pain. Studies suggest that the widespread and unspecific nature of hypersensitivity in CTTH indicates that general pain sensitivity is affected at the central level [5]. In case of migraine, pericranial tenderness increases with attack frequency as well [97]. However, there is no difference in the mechanical pain threshold between individuals with chronic migraine during or outside the headache phase [98], or in the mechanical and thermal pain thresholds between episodic and chronic migraine [99]. These findings raise questions about whether central sensitization may explain the pericranial tenderness in migraine or if the tenderness is merely a secondary phenomenon of migraine attacks. In migraine, cephalic cutaneous allodynia is believed to be due to the sensitization of second-order neurons in the caudal trigeminal nucleus, while extracephalic cutaneous allodynia may be due to the sensitization of third-order thalamic neurons [55, 69].

Peripheral nociceptive fibers, originating from the C2 dorsal root ganglion, traverse the occipital muscles before innervating the posterior dura, suggesting a functional connection between intra- and extracranial structures [100]. This suggests that activation of the posterior dural nociceptors may stimulate the onset of headache and muscle tenderness instead of via the convergence of the dural and muscle nociceptors in the trigeminocervical complex [100]. Biopsies from the neck periosteum in individuals with chronic migraine and neck pain have shown increased expression of pro-inflammatory genes and decreased expression of anti-inflammatory genes [101], indicating the involvement of inflammation as a possible underlying cause of neck pain in migraine, in addition to pericranial tenderness. [91]. Studies in preclinical mouse models have shown that potential local inflammation in neck muscles leads to sensitization of muscle nociceptors with increased cytokine levels and subsequent activation of sensory afferents [102]. In individuals with frequent ETTH, increased pain sensitivity has been reported after intramuscular infusion of inflammatory substances, suggesting the onset of peripheral and central sensitization in TTH [5]. There are no consistent data on altered cytokine levels in TTH [5], while studies in migraine suggest the potential involvement of altered cytokine levels [103]. While both TTH and migraine involve pericranial muscles and myofascial factors, they differ in terms of the underlying pathways. In migraine, activation and sensitization of the first-order trigeminovascular neurons and vascular input likely play a greater role, whereas in TTH, peripheral muscular factors may be more involved, leading to central sensitization and the development of CTTH. Thus, central sensitization plays a role in both migraine and TTH. Furthermore, the central sensitization mechanism is involved in the pathophysiology of fibromyalgia [41]. This mechanism involves the impact of neuromediators on nociceptive stimulation within the central nervous system, leading to a decrease in pain threshold to various stimuli not only in localized painful areas but also in non-painful body parts [41]. Individuals with chronic headache may also exhibit heightened sensitivity to pain beyond the cephalic region [41, 69, 104]. Therefore, headache may play a role as a triggering factor for fibromyalgia in patients with migraine, TTH and fibromyalgia [41, 105, 106].

#### *Neuropeptides*

Experimental studies have shown that neuropeptides such as CGRP, pituitary adenylate-cyclase-activating polypeptide (PACAP)-38, and nitric oxide (NO)/GTN are involved in the pathophysiology of migraine [59, 85, 107]. Upon exposure to these neuropeptides, individuals with migraine develop migraine attacks, while healthy participants only report mild or no headache [59, 85]. The cAMP (mediated by CGRP and PACAP-38) and cGMP pathways (mediated by NO) and ATP-sensitive potassium (KATP) channels have been suggested to be involved in the pathophysiology of migraine [59, 85]. However, there is no change in peripheral or cranial blood or CSF levels of CGRP nor peripheral or cranial blood levels of vasoactive intestinal peptide (VIP), substance P, or neuropeptide Y in TTH, suggesting less or no prominent involvement of these peptides in the TTH pathophysiology [5, 108].

#### *Nitric oxide*

NO mechanisms appear to be involved in both migraine and TTH. GTN, a nitric oxide donor, induces migraine attacks in ​​ ~ 90% of individuals with migraine [85, 109, 110]. In one study with individuals with CTTH, after exposure to exposed to NO, the majority of individuals (~ 90%) developed TTH, while only two patients experienced an attack classified as migraine without aura [111]. These two patients described their usual TTH as both pressing and throbbing in quality, and moderate in intensity. Only a few healthy controls experienced a delayed headache after GTN, which was mainly mild in intensity, as opposed to the more pronounced headache in TTH [111]. The time to peak median headache intensity in TTH was 8 h after GTN infusion with a median pain score of 4 out of 10 [111]. In migraine, the time to peak headache intensity was earlier at 5.5 h post-infusion with a median pain score of 3 out of 10 [110]. Inhibition of nitric oxide synthase (NOS) effectively aborts migraine attacks [107, 112], and reduces the pain intensity of headache in CTTH compared to placebo [113]. NO may play a role in the development of central sensitization of nociceptive pathways, and it is likely that inhibition of NOS results in the reduction of central sensitization [113]. However, the role of NOS in chronic migraine patients has not been studied yet.

#### *Serotonin*

In CTTH, the serotonin (5-HT) levels in platelets and plasma are normal or decreased, with a normal platelet 5-HT uptake compared with controls [114, 115]. In contrast, the serotonin levels are increased in ETTH with a decreased platelet 5-HT uptake in ETTH [116]. Platelet 5-HT decreases and 5-HIAA, a primary 5-HT metabolite, increases during migraine attacks as in CTTH [117]. This suggests the involvement of serotonergic mechanisms in both migraine and TTH. Descending inhibitory pathways involve the release of 5-HT [118]. One of the rich-5-HT nuclei, the nucleus raphe magnus (NRM), is the origin of the descending serotonergic pathways, which play a role in the pathophysiology of both CTTH and migraine [5].

### Pharmacological treatment

The treatment of primary headaches, including migraine and TTH, involves both non-pharmacological and pharmacological interventions [5]. Pharmacological management for these headaches can be divided into acute and preventive treatment. In the case of migraine, a stepped-care approach is recommended using three lines of acute medications [119, 120]. The first line of medications includes NSAIDs (such as aspirin, ibuprofen, or diclofenac) and simple analgesics (such as paracetamol/acetaminophen) for those intolerant to NSAIDs [55, 59, 121]. For TTH, the pharmacologic approach to acute headache episodes remains disease non-specific. Simple NSAIDs and acetaminophen are generally safe, economical, and efficacious pharmacologic treatments for TTH as in the acute treatment of migraine [11]. The European Federation of Neurological Societies Task Force (EFNS-TF) recommends the use of multiple oral NSAIDs in the treatment of TTH [122]. Among NSAIDs, ibuprofen and ketoprofen have demonstrated the highest efficacy in the treatment of TTH [16]. Furthermore, caffeine combination therapies have demonstrated improved efficacy in TTH with minimal alteration to the previous safety profile [123].

According to the step-care approach, triptans are considered second-line medications for the acute treatment of migraine [124]. Randomized controlled trials (RCTs) have shown that triptans are effective in reducing the intensity and duration of migraine headaches and improving other migraine-related symptoms, such as nausea, vomiting, and sensitivity to light and sound [125]. In one study, sumatriptan was found to have an effect on chronic tension-type headache (CTTH) compared to placebo [126]. However, the small sample size, coexistence with migraine, or central sensitization effect may limit the power of the study. Limited evidence suggests that triptans are not effective for tension-type headaches (TTH) and are not recommended [58, 122, 127].

If triptans fail or are contraindicated, the third-line medications for acute treatment of migraine include newer drugs such as gepants and ditan (lasmiditan) [128]. However, at present time, these novel medications are not yet available in many countries and have not been studied in other primary headache disorders [120].

#### *Preventive treatment*

Traditional migraine preventive medications are non-specific and were initially intended for other indications. The most frequently used are antihypertensive, antidepressant, anticonvulsive agents, and calcium-channel blockers. Onabotulinumtoxin A is effective in chronic migraine but not in chronic TTH [122]. However, more recently, a new class of drugs has been specifically developed for migraine prevention: calcitonin gene-related peptide (CGRP) monoclonal antibodies (mAbs) targeting the CGRP or its receptor, as well as oral CGRP receptor antagonists [128].

In contrast, the preventative pharmacologic approach to TTH remains disease non-specific. Preventative pharmacologic treatments for TTH are recommended only for patients diagnosed with frequent ETTH and CTTH [5, 6]. Three medications are currently recommended by the EFNS-TF for the preventative treatment of CTTH: amitriptyline, mirtazapine, and venlafaxine. These medications have proven effects on the management of CTTH [5]. Of these, amitriptyline is supported by the highest level of evidence, but also a high frequency of adverse events [129]. Of note, amitriptyline and venlafaxine have been shown to be effective in preventing migraine in clinical trials [127, 128]. These drugs are commonly used as first-line treatments for chronic migraine prevention, with amitriptyline being supported by the highest level of evidence [127, 128]. Alternative medications such as tetracyclic and atypical antidepressants are supported by a lower level of evidence for TTH prevention and are primarily reserved for patients that have reported severe adverse events or poor initial efficacy to the aforementioned therapies [5, 122]. Amitriptyline and venlafaxine have also been proven to be effective in the prevention of migraine.

### Non-pharmacological treatment

The non-pharmacological interventions can have varying degrees of impact on headache symptoms, disability, and the quality of life in both TTH and migraine (Table 4). Acupuncture has been found to provide improvements in acute medication for TTH and headache intensity and frequency for migraine [130–133]. Studies on exercise interventions are more limited for TTH, but aerobic, strengthening, and stretching exercises have shown potential benefits in reducing headache duration, pain intensity, frequency, disability, quality of life, and psychological variables for both TTH and migraine [130, 134–136]. Trigger point therapy and manual therapy can also be effective in reducing headache symptoms, with trigger point therapy reducing intensity and manual therapy reducing the duration and frequency of headaches in TTH and migraine [137–139]. Physical therapy interventions such as transcutaneous electrical nerve stimulation (TENS), massage, and cold application, as well as neuromodulation applications, may also be beneficial for both TTH and migraine [140–143]. Psychological treatments [144–148] and biofeedback [149–151] can help to regulate pain pathways and improve the quality of life for both types of headaches, while diet modifications may be useful in identifying trigger foods and reducing headache severity [152–154]. It is not known whether one treatment is superior to others in either TTH or migraine, and there is a lack of evidence about the optimal duration, frequency, and number of sessions for non-pharmacological approaches [134]. Patient preferences for drug side effects or non-pharmacological approaches may be an important consideration [155]. While active physical therapy approaches have not shown negative effects, more comprehensive and long-term relaxation therapy may be required for psychological treatments [137, 156]. Further randomized controlled studies are needed for neuromodulation applications in both types of headaches [143].

**Table 4:** Similarities and differences in treatment responses between TTH and migraine for non-pharmacological approaches

	**TTH**	**Migraine**
Acupuncture	Medication use ↓	Headache intensity ↓ (Epis., Chr.) Headache days ↓ (Epis., Chr.)
Aerobic Exercise	Headache duration ↓ Disability ↓ Quality of Life ­↑ Psychological well-being ­↑	Headache intensity ↓ (8–12 weeks) Headache frequency ↓ Headache duration ↓ (8–12 weeks) Medication use v (12 weeks) Disability ↓ Quality of Life ­↑ Psychological well-being ­↑
Strengthening Exercise	Headache intensity ↓ (3 weeks-3 months)	Headache frequency ↓
Cranio-cervical Exercises	Headache duration ↓ (6 weeks) Medication use ↓ (6 weeks)	Disability ↓ Neck Muscle performance ­↑
Stretching Exercise	Headache frequency ↓ (6 weeks)	Headache frequency ↓
Trigger Point Therapy	Headache intensity ↓	Not clear
Manuel Therapy	Monthly headache days ↓ Headache duration ↓	Quality of Life ↑
Spinal Manipulative Treatment	Headache intensity ↓ (6 weeks) (Chr.) Headache frequency ↓ (6 weeks) (Chr.) Medication use ↓ (6 weeks) (Chr.) Functional well-being ↑ (Chr.)	Headache intensity ↓ Migraine days ↓
Mixed Physical Therapy	Headache intensity ↓ (TENS, Massage, Coldpack) Muscle sensitivity ↓ (TENS, Massage, Coldpack) Headache intensity ↓ (Physical therapy + Craniocervical exercises) Headache frequency ↓ (Physical therapy + Craniocervical exercises)	Headache frequency ↓ (Diaphragm training + Cervical mobilization + Massage + Myofascial release + Trigger Point + Passive stretch + Medications)
	Headache duration ↓ (Physical therapy + Craniocervical exercises)	
Neuromodulation Treatment	Headache intensity ↓ Quality of Life ­↑	Monthly headache days ↓ Headache duration ↓ Medication use ↓
Psychological Treatment	Headache frequency ↓ Disability ↓	Headache intensity ↓ Headache duration ↓ Disability ↓ Quality of Life ­↑
Biofeedback	Headache frequency ↓	Headache frequency ↓
Diet	Low-fat diet	Low-fat diet

*TENS* Transcuteneous electric nerve stimulation

### Lessons learned and future directions

The studies suggest that TTH and migraine are distinct primary headache disorders, despite some similarities in their clinical presentation. One major challenge in diagnosing these headaches is the lack of available biomarkers, and diagnosis is largely based on criteria and self-reporting by patients While a headache diary can be helpful, it is not optimal, and future research should explore potential biomarkers, including imaging biomarkers. Clinical distinctions between TTH and migraine include the lack of accompanying autonomic symptomatology in TTH, as well as accompanying symptoms of photophobia, phonophobia, and nausea, and worsening of headache upon routine physical activity more specific to migraine than TTH [5, 83]. TTH episodes may continue for up to 7 days, while individual migraine episodes do not last longer than 3 days, although the mechanisms involved in the termination of both types of headaches remain unknown [5]. Despite these clinical differences, TTH is often misdiagnosed as migraine, and both types of headaches are commonly underdiagnosed [3, 5, 157]. The prevalence and characteristics of TTH in individuals with coexistent migraine are similar to the general population, but TTH attacks are more severe in intensity [8]. The concurrence of migraine and TTH may occur by chance, but further investigation is needed to determine if there is a causal mechanistic relationship between the two disorders [5].

Epidemiological studies are needed to better distinguish these headaches and control for common covariate variables, such as medication overuse, age, sex, and psychiatric comorbidities. Both TTH and migraine are common primary headache disorders with a substantial disease burden, with migraine ranking at the top in terms of years lived with disability [5, 17, 157]. While current research shows a similar prevalence of migraine and TTH in children, with no difference between boys and girls, more data are needed in these age groups. From age 20–64, there is a difference in sex distribution, as migraine becomes more prevalent in females as opposed to the more equal distribution of TTH in the same age group. These data altogether further support the notion of stronger hormonal influences on migraine compared to TTH. Further investigation into this aspect of headache is needed. While both are associated with genetic factors, specific genes responsible for TTH's heritable nature remain unknown, in contrast to multiple risk loci associated with migraine [5, 85]. While muscular factors appear to play a more important role in TTH than in migraine, additional studies are needed to further explore the involvement of muscles in TTH. Recent research has demonstrated the role of neuropeptides, including CGRP, in the pathophysiology of migraine. Additionally, novel targets of interest include pituitary adenylate cyclase-activating polypeptide 38 (PACAP-38), adrenomedullin, and multiple transient receptor potential (TRP) channels. It should be recognized that significantly less is known regarding the cellular and molecular explanation of TTH. Although pharmacological provocation studies have provided valuable information leading to the discovery of the aforementioned targets of migraine pathophysiology, these results have not been thoroughly investigated in TTH. Interestingly, nitric oxide (NO) mechanisms might be involved in both migraine and TTH, which needs further investigation. Allodynia, a marker of central sensitization, has been observed in both CTTH and migraine, highlighting a potential commonality between the two conditions (164, 165). While the trigeminovascular system is implicated in both TTH and migraine, the activation and processing of nociceptive input seem to differ between the two conditions. Further research is needed to elucidate the mechanisms underlying nociceptive input in both TTH and migraine. Both neck pain and pericranial tenderness are highly prevalent in both migraine and tension-type headache (TTH), challenging the notion that the onset of TTH pain is primarily through the trigeminocervical complex compared to migraine. While vascular input in TTH appears to be of lesser significance than in migraine, further data are required to confirm this observation. In contrast, the increased risk of stroke and myocardial infarction in migraine patients supports the involvement of vascular factors in migraine rather than TTH.

Several treatments have been shown to be effective for migraine but not TTH, including onabotulinumtoxinA, antihypertensive preventatives, and triptan. NSAIDs are commonly used to treat both TTH and migraine patients; however, no additional medications have demonstrated efficacy in TTH [5]. Several novel molecular entities are undergoing clinical trials for efficacy in migraine, with a particular interest in prophylaxis. The entities of interest include CGRP and PACAP-38 targeting drugs, and drugs that target specific TRP channels.

The role of non-responsive patients remains debatable and poses the question of the benefits in further subdividing clinical cohorts by the underlying molecular mechanisms of their individual migraine attacks. Unfortunately, due to the lack of current therapeutic targets, this approach is not yet feasible for TTH patients. 

## Data Availability

There is no data available in the article, but the corresponding author can be contacted if more information is requested.
